# Deadtime effects in quantification of ^177^Lu activity for radionuclide therapy

**DOI:** 10.1186/s40658-017-0202-7

**Published:** 2018-01-11

**Authors:** Carlos F. Uribe, Pedro L. Esquinas, Marjorie Gonzalez, Wei Zhao, Jesse Tanguay, Anna Celler

**Affiliations:** 1 Medical Imaging Research Group, University of British Columbia, Vancouver, BC Canada; 2 Department of Molecular Oncology, British Columbia Cancer Research Centre, Vancouver, BC Canada; 3 Vancouver Coastal Health Authority, Vancouver, BC Canada

**Keywords:** ^177^Lu, Deadtime, SPECT/CT, Quantification

## Abstract

**Background:**

The aim of this study was to investigate the deadtime (DT) effects that are present in ^177^Lu images acquired after radionuclide therapy injection, assess differences in DT based on the full spectrum and the photopeak-only measurements, and design a method to correct for the deadtime losses.

A Siemens SymbiaT SPECT/CT camera with a medium energy collimator was used. A 295-mL bottle was placed off-center inside a large cylinder filled with water, and ^177^Lu activity was sequentially added up to a maximum of 9.12 GBq. The true count rates vs. observed count rates were plotted and fitted to the DT paralyzable model. This analysis was performed using counts recorded in the full spectrum and in other energy windows. The DT correction factors were calculated using the percentage difference between the true and the observed count rates.

**Results:**

The DT values of 5.99 ± 0.02 μs, 4.60 ± 0.052 μs, and 0.19 ± 0.18 μs were obtained for the primary photons (PP) recorded in the 113- and 208-keV photopeaks and for the full spectrum, respectively. For the investigated range of count rates, the DT correction factors of up to 23% were observed for PP corresponding to the 113-keV photopeak, while for the 208-keV photopeak values of up to 20% were obtained. These values were almost three times higher than the deadtime correction factors derived from the full spectrum.

**Conclusions:**

The paralyzable model showed to be appropriate for the investigated range of counts, which were five to six times higher than those observed in the patient post-therapy imaging. Our results suggest that the deadtime corrections should be based on count losses in the scatter-corrected photopeak window and not on the deadtime determined from the full spectrum. Finally, a general procedure that can be followed to correct patient images for deadtime is presented.

## Background

Peptide receptor radionuclide therapies (PRRT) using ^177^Lu*-*labeled somatostatin analogues have been proven to be very effective in treatment of neuroendocrine tumors (NETs), taking advantage of the overexpression of somatostatin receptors in NET cancer cells [1–3]. A limitation on how this treatment is delivered in many centers is that it does not take into account differences in radiotracer uptake between individuals and all patients are injected with the same activity [4]. It is believed that treatment plans using injections based on an individualized dose assessment, similar to what is done in external beam therapies, could significantly improve PRRT outcomes and therefore should become routine practice [5, 6].

In order to achieve such personalized dose assessment, the accurate quantification of activity within organs of interest (critical organs) and tumors must be performed and temporal changes of this activity determined. In typical diagnostic imaging scans, the administered activities are low, resulting in low count rates in the SPECT camera with no deadtime (DT). However, in radionuclide therapy procedures, as those performed with ^177^Lu*,* patients are injected with high activities (of the order of GBq), which result in high photon flux and may cause camera DT when imaging studies are performed. In order to accurately quantify patient’s activity in these situations, correcting images for DT losses might be necessary.

Several studies have investigated DT effects in Anger cameras using high activities of ^99m^Tc and ^131^I [7–13]. However, to the best of our knowledge, only two studies have examined this effect for ^177^Lu*.* Beauregard et al. [14] investigated DT effects using phantom acquisitions and image quantification protocol in which the dual energy window (DEW) scatter correction method was applied. The effects of DT on the counts recorded in the full spectrum and in the photopeak window were measured, and the data were fitted to the Sorenson’s paralyzable model [8]. The authors designed a correction scheme where they created lookup tables relating the DT corrections values for the reconstructed image with the count rates observed in the full energy spectrum.

Celler et al. [15] proposed a marker-based method for the determination of the DT correction. The counts losses in the image of a small marker placed in the field of view (FOV) of the camera and imaged simultaneously with the patient, relative to the same marker counts without the patient, were used to determine the DT correction factor. This procedure, however, is quite cumbersome, and tests were performed for low-energy high-resolution (LEHR) collimator which presents additional challenges (need to account for high scatter and septal penetration) relative to the typically recommended medium-energy (ME) collimator [14, 16, 17].

The aim of our study was to investigate the DT effects that are present in imaging studies of the ^177^Lu radionuclide therapy patients, performed according to the guidelines outlined in MIRD 26 [18]. Our objective was to design an accurate DT correction method to be used in these studies.

Since quantification of activity for dosimetry purposes is done using images reconstructed from primary photons (PP) recorded in the photopeak window(s), the deadtime correction method must correct for count losses that affect only these primary photopeak photons. Therefore, the proposed DT correction method is based on the analysis of primary counts, i.e., counts that are collected in the photopeak window(s) and have the scatter/background component(s) removed. Additionally, we assessed differences between the DT values and the correction factors determined using our primary photon-based method for 113-keV, 208-keV photopeaks and those obtained from the analysis of count loses in the full spectrum.

A similar study to ours was performed by Guy et al. [13], but they used ^131^I while in our case, it is ^177^Lu. They found large differences between the deadtime correction factor which was based on the analysis of the primary photon count losses and that based on the count losses in the full spectrum. The full-spectrum correction factor would be underestimated by up to 20% when compared to the DT correction based only on PP.

Sorenson [8] suggested that gamma cameras behave as having a combination of paralyzable and non-paralyzable components. Several studies from the second half of the last century [7, 19] have found that, at least for the range of count rates encountered in medical procedures, the camera behavior could be accurately approximated by a paralyzable model. More recently, Silosky et al. [12] analyzed modern cameras and also concluded that for count rates below 375 kcps, the cameras behaved as paralyzable systems. Guirado et al. [11] found that for a Symbia camera (Siemens Medical, Germany), a sharp change in camera response can be observed at very high count rates, but in the region below this change, both paralyzable and non-paralyzable models accurately fit the data. A study by Guy et al. [13] suggests that modern cameras are dominated by the paralyzable component.

Based on our experience with patients treated by our collaborators at the *L’Hôtel-Dieu de Québec* site of the *CHU de Québec – Université Laval* center (Quebec City, Canada), the total count rates observed (full spectrum) during the first imaging scan after the therapeutic ^177^Lu injection (which occurs between 1 and 4 h after the injection) are typically of the order of 70–100 kcps. This corresponds to the low range of the count rates observed in the experiments performed in this study, which for the full spectrum, were equal to about 400 kcps.

## Methods

### SPECT camera, collimators, and energy window setup

The experiments were performed using a Siemens SymbiaT (Siemens Medical, Germany) SPECT/CT camera with a medium-energy low-penetration (MELP) collimator. Two photopeak energy windows (PW) were specified; one for the 113-keV and another one for the 208-keV photopeaks of ^177^Lu (Table 1). Additionally, lower scatter window (LSW) and upper scatter window (USW) were defined for each of the photopeaks (four in total) to be used in triple energy window (TEW) scatter correction [18]. We have been using such three-energy-window acquisitions in all our research and clinical studies, and this approach has been shown to lead to accurate activity quantification [20]. Furthermore, the counts in the full energy spectrum (i.e., 0 keV to 400 keV) were also collected.

**Table 1:** Energy window limits for the two photopeaks of ^177^Lu. Additional energy windows were used to collect data for the entire spectrum

Window name	Lower limit [keV]	Center [keV]	Upper limit [keV]
LSW_113_	88.4	95.0	101.7
PW_113_	101.7	113.0	124.3
USW_113_	125.0	139.0	152.9
LSW_208_	153.0	170.0	187.0
PW_208_	187.2	208.0	228.8
USW_208_	229.5	255.0	280.5

### Planar acquisitions

**Fig. 1:**
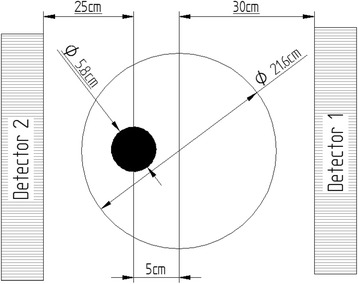
Coronal view of the positioning of the phantom and the bottle inside it with respect to the two detectors

In order to measure the camera DT, its response has to be determined over a wide range of activities. A total of 9*.*12 ± 0*.*91 GBq of ^177^Lu (in the form of ^177^LuCl_3_, obtained from Polatom, Poland) *was* diluted in water to obtain a solution with a concentration of 35*.*7 ± 1*.*4 MBq/mL and distributed equally into twenty-five 10-mL syringes. The activity contained in each syringe was measured using an Atomlab100 plus (Biodex, USA) dose calibrator. The syringes were sequentially emptied into a 295-mL bottle located 5 cm off-center inside a large cylinder (21.6-cm diameter) filled with water. The cylinder was placed on the camera bed between the detectors, so that the center of the bottle was positioned at 35 and 25 cm from the collimator surface of detector 1 and detector 2, respectively (see Fig. 1). Since the routine quality control tests of the camera ensure that the behavior of both detectors is very similar, the 10 cm of additional water thickness for detector 1 allowed us to investigate the effects of attenuation and scatter on the count rate and camera DT. Activity residues in each emptied syringe were measured again in the dose calibrator and the net activity which was added to the bottle was calculated for all 25 syringes.

A series of 26 planar scans was performed. In order to determine background counts, the first planar scan was performed with no activity in the bottle. The subsequent scans were performed after emptying each consecutive syringe into the bottle. The acquisition time of the scans varied from 3 min for very high activity to 5 min for lower activity points.

Additionally, the energy spectra were collected for both detectors at low (17.3 kcps), medium (101.5 kcps), and high (306.3 kcps) count rates.

### Data analysis

In the analysis of the DT effects, the counts collected over the entire field of view (FOV) of each of the detectors were used and the corresponding count rates were obtained by dividing these counts by the scan acquisition times.

Since we did not see any sharp changes in the camera behavior (mentioned by Guirado et al. [11]) and a similar work by Guy et al. [13] also suggested that modern cameras follow a paralyzable model, we decided to fit our data to the Sorenson’s paralyzable model [8] using the following equation:

1 $$ {R}_o={R}_t{e}^{-{R}_t\tau } $$

where *R*_*t*_ represents the true count rate, *R*_*o*_ is the observed count rate obtained from the experiment, and *τ* is the paralyzable DT parameter. In order to estimate the true count rates *R*_*t*_ in each of the analyzed energy windows, a linear fit was made to the data points corresponding to acquisitions where the activity in the phantom was lower than 2.1 GBq (< 80 kcps). We assumed that no deadtime was present at these low count rates. The parameter *τ* was determined by fitting the data to Eq. (1) using the total least square algorithm.

**Fig. 2:**
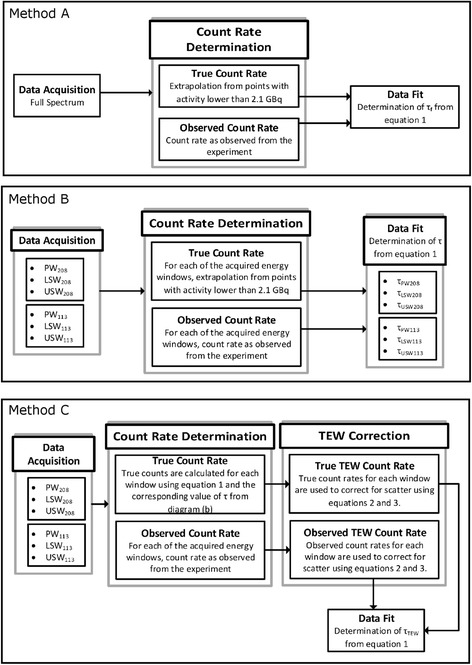
Flow charts of the three methods used to calculate the camera deadtime

In the analysis of the DT effects for each detector, the following three methods were used (summarized in Fig. 2):

A. DT estimated using count rates in the full spectrum

For each acquisition, the observed and the true count rates for photons recorded in the full energy spectrum were used to determine the value of *τ*_*f*_. True count rates were estimated using the extrapolation method previously described.

B. DT estimated for counts acquired in each energy window

In order to compare the photopeak window DT values with the primary photons DT (obtained after correcting PW photons for scatter using TEW), the observed (measured) and the true (obtained from extrapolation) count rates were determined independently for each of the two photopeak windows (PW_113_ and PW_208_) and all four scatter windows (LSW_113_, USW_113_, LSW_208_, and USW_208_). For each energy window, the corresponding DT values (i.e., ${\tau}_{\mathrm{P}{\mathrm{W}}_{208}},{\tau}_{\mathrm{P}{\mathrm{W}}_{113}},{\tau}_{\mathrm{LS}{\mathrm{W}}_{208}},{\tau}_{\mathrm{LS}{\mathrm{W}}_{113}},{\tau}_{\mathrm{US}{\mathrm{W}}_{208}},{\tau}_{\mathrm{US}{\mathrm{W}}_{113}}\Big)$ were independently calculated, in a similar way as done for the full spectrum.

C. DT estimated for primary photons count losses

As already mentioned, the activity quantification is done using images reconstructed from primary photons only. In order to determine the number of primary photons (and primary photons count rate) detected during each of the 26 scans in the two photopeak windows, the TEW correction method was applied. The method estimates the scattered counts (*C*_*s*_) collected in each of the PW windows from the counts collected in the corresponding LSW (*C*_*ls*_) and USW (*C*_*us*_) using Eq. (2).

2 $$ {C}_{\mathrm{s}}=\left(\frac{C_{\mathrm{ls}}}{\omega_{\mathrm{ls}}}+\frac{C_{\mathrm{us}}}{\omega_{\mathrm{us}}}\right)\frac{\omega_{\mathrm{pw}}}{2} $$

The widths of the LSW, PW, and USW are given by *ω*_ls_, *ω*_pw_, and *ω*_us_, respectively. The primary counts (*C*_prim_) for the two photopeaks (113 and 208 keV) are calculated by removing scattered counts from the total counts recorded in the corresponding photopeak window (*C*_PW_).

3 $$ {C}_{\mathrm{prim}}={C}_{\mathrm{PW}}-{C}_{\mathrm{s}} $$

Count rates are calculated by dividing (*C*_prim_) by the duration of each scan.

A similar procedure as described in methods A and B was followed to determine the DT values for primary photons only. First, the true and observed count rates, for each of the six energy windows, were determined (method B). The observed count rates were measured experimentally, while the true count rates for each energy window were calculated using Eq. 1 and the corresponding value of *τ* (determined in Method B). Alternatively, the true count rates can be estimated by extrapolation, as in method B. Next, these observed and true count rates were separately entered into Eq. 2 to determine the observed and the true scatter components. Finally, the observed and the true primary photons count rates were estimated using Eq. 3.

In the last step, the observed and true primary photons count rates were fitted to Eq. 1 to obtain the primary photons DT value *τ*_PP_ for each of the two photopeaks.

With the known camera deadtime values, it is possible to calculate deadtime correction factors (DTCFs) for the full spectrum, for each of the energy windows, and for the primary photons only. DTCF corresponding to any particular observed count rate can be calculated as the percentage difference between the true and the observed count rates:

4 $$ \mathrm{DTCF}=\frac{R_t-{R}_o}{R_t}\times 100 $$

To perform the correction, the values of the count rates observed in the experiment (or patient scan) should be used, while the values of the true count rates can be calculated using Eq. 1 and the corresponding *τ* values. These *τ* values must be determined experimentally using phantoms.

## Results

**Fig. 3:**
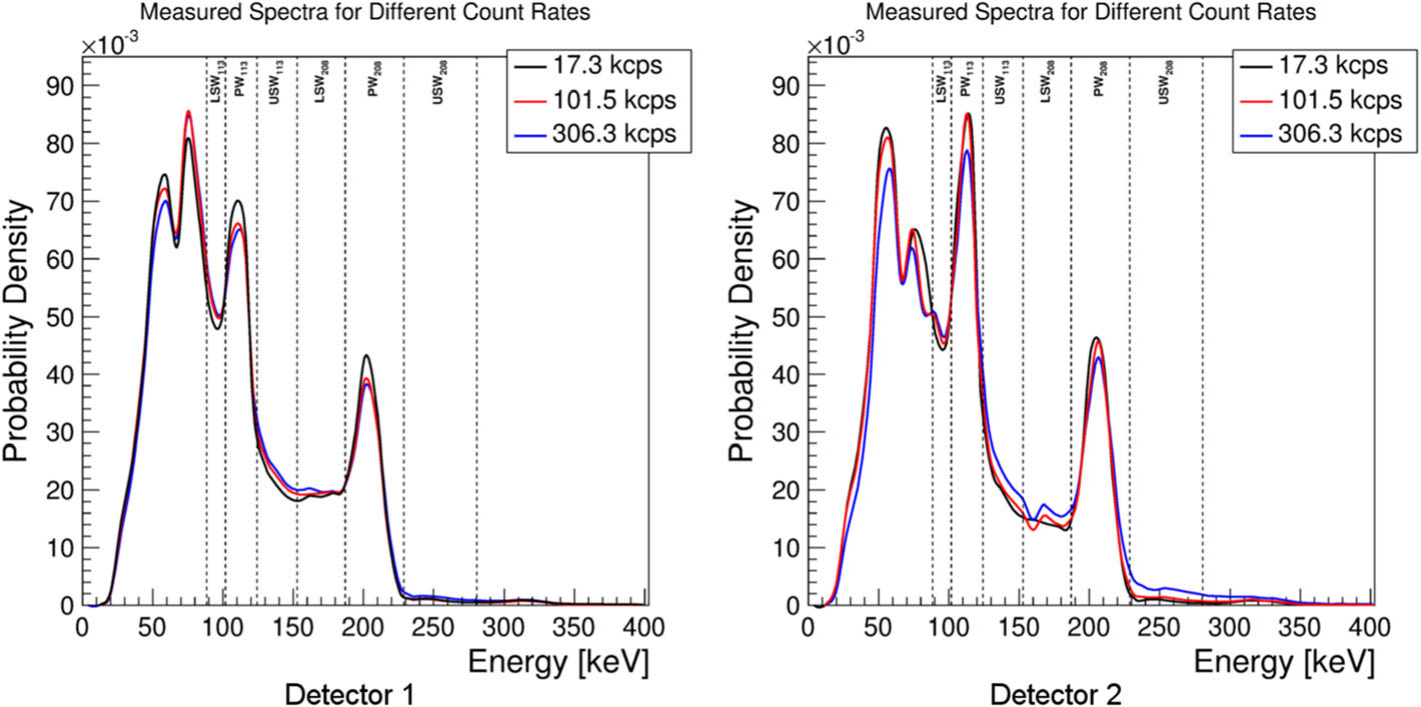
Measured spectra recorded at different count rates for both detectors. The count rates listed in the legend of the plots correspond to the total full spectrum count rates

Figure 3 shows the spectra collected for both detectors at low, medium, and high count rates. For display purposes, the spectra were normalized so that the total area under the curve was equal to one (i.e., probability density functions). In reality, due to different attenuation conditions, the number of counts collected by detector 1 was about five times lower than that in detector 2. The vertical dashed lines show the positions of the energy windows.

**Fig. 4:**
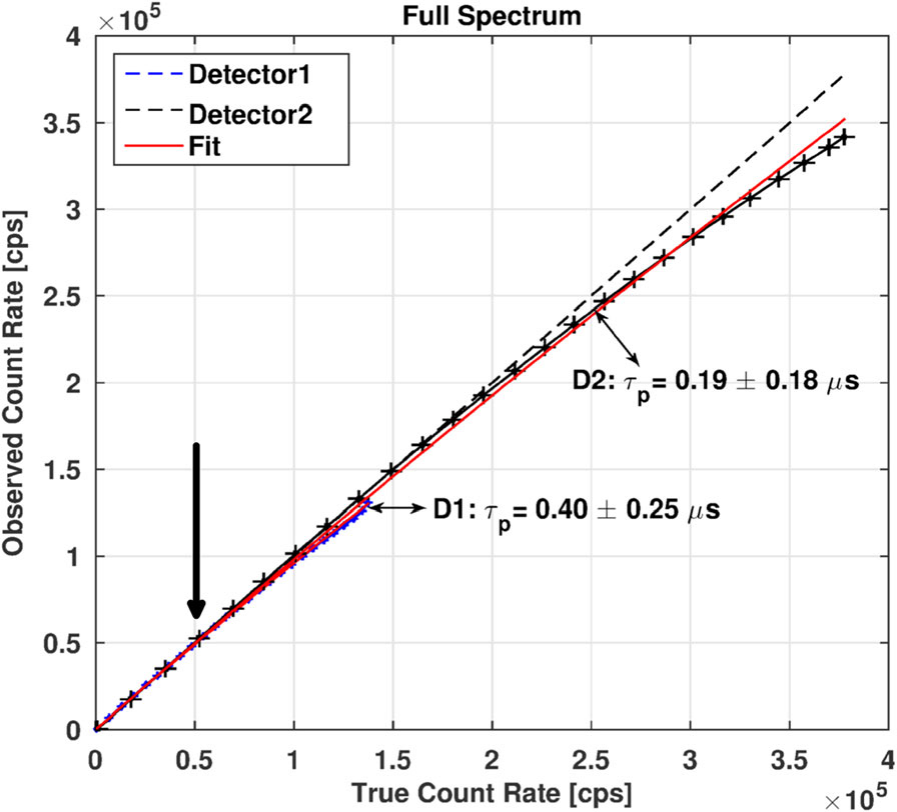
Observed count rates vs. true count rates for the full spectrum. The values of *τ*_p_ determined for both detectors are shown. Crosses indicate the measured data points. Typical values of full spectrum count rates in patient studies (indicated by an arrow) range from 50 to 70 kcps (~ 0.6 × 10^5^ cps on this graph)

**Fig. 5:**
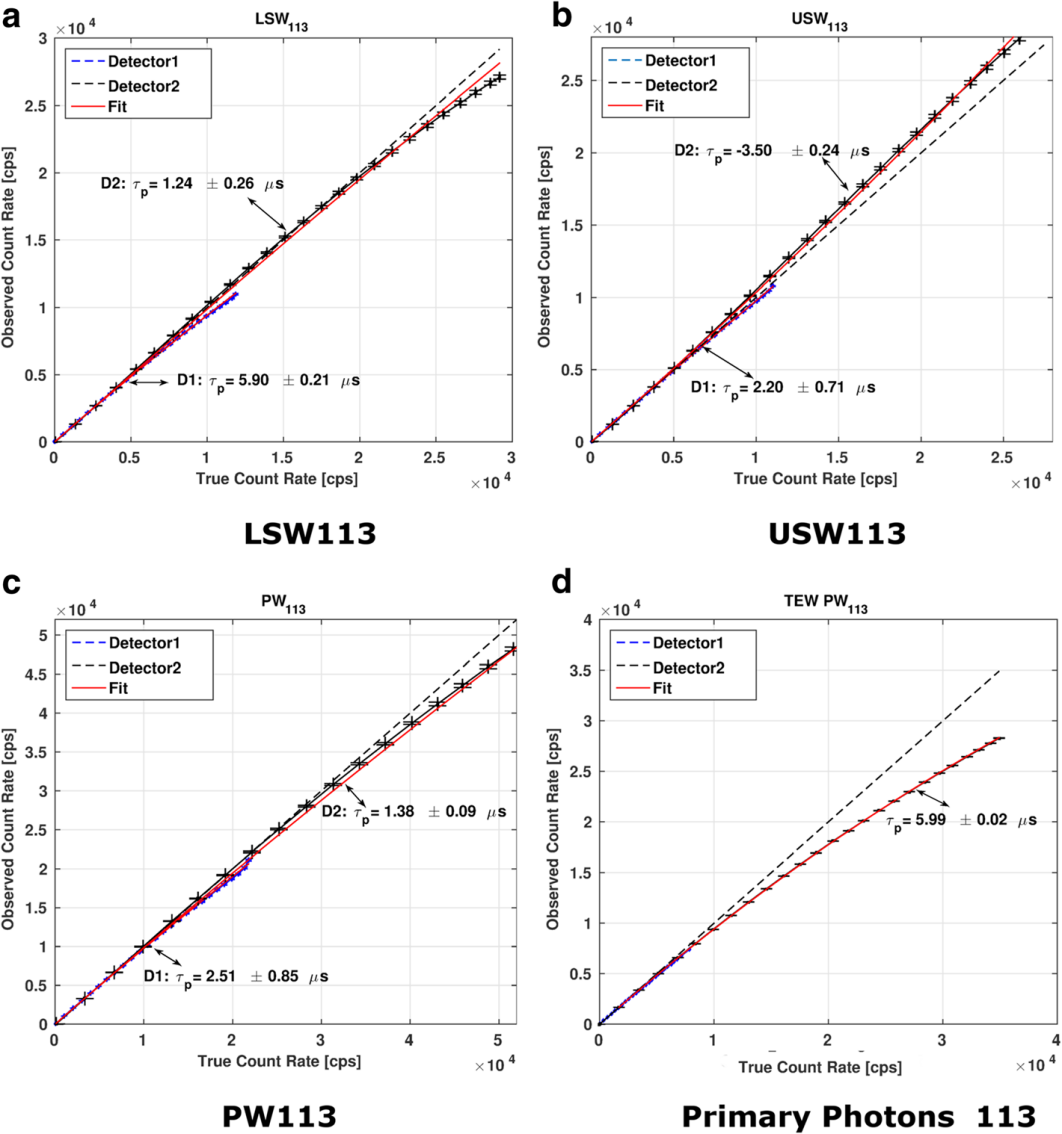
Observed count rates as a function of true count rates for the 113-keV photopeak (**a**) and its scatter windows (**b**, **c**). The TEW scatter corrected behavior for this photopeak is shown in (**d**)

**Fig. 6:**
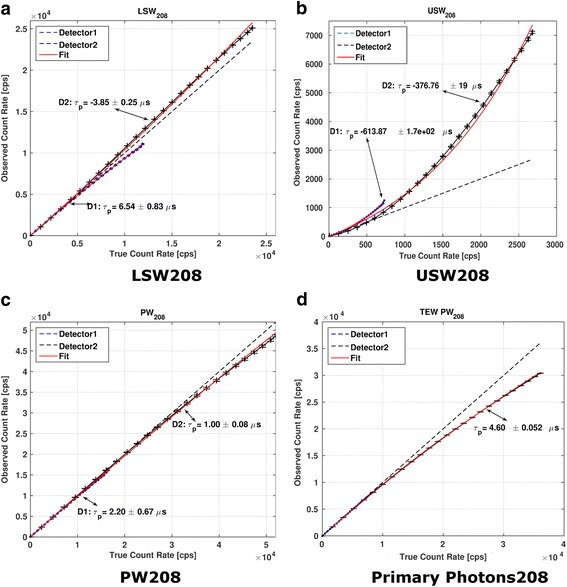
Observed count rates as a function of true count rates for the 208-keV photopeak (**a**) and its scatter windows (**b**, **c**). The TEW scatter corrected behavior for this photopeak is shown in (**d**)

The behavior of the observed count rates vs. true count rates for the full spectrum is shown in Fig. 4. Plots of observed count rates vs. true count rates for the LSW, USW, and PW and for the primary photons corresponding to the 113 and 208 keV photopeaks of ^177^Lu are shown in Figs. 5 and 6, respectively. The curves for each detector have been plotted separately. The dashed lines represent the identity lines which correspond to the case where no DT occurs. All the symbols in blue show the data for detector 1, while those in black are for detector 2. The fit to the paralyzable model is displayed in red.

In the case of detector 2, for which larger range of count rates than in detector 1 were observed (due to less attenuation), the values of DT determined from PW_113_ and PW_208_ exceeded the DT values determined from the full spectrum by a factor of 7.3 and 5.3, respectively. When considering only primary photons (with the TEW scatter correction applied), the DT values exceed those obtained from the full spectrum by a factor of 31.5 and 24.2 for the 113 and 208 keV, respectively. For all the fits, the coefficients of determination (*R*^2^) were higher than 0.95 suggesting a good fit of the observed count rates to the paralyzable model.

**Fig. 7:**
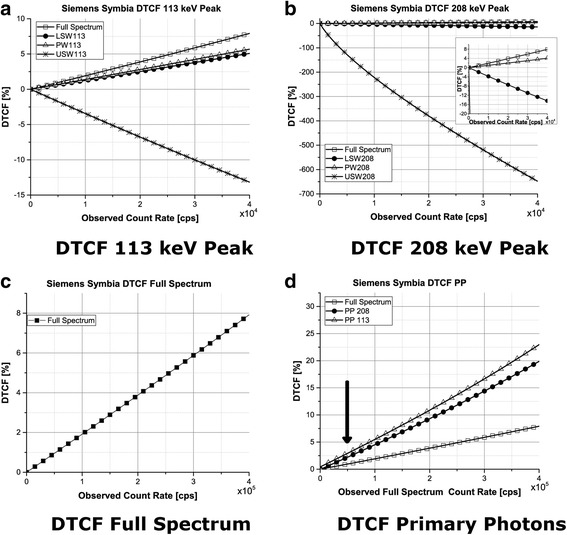
Deadtime correction factors as a function of the observed count rate. For figures **a**, **b**, and **c**, the observed count rates are those which were recorded in the energy window corresponding to the presented curve (see legend). **d** DTCF corresponding to PP. Typical values of full spectrum count rates in patient studies (indicated by an arrow) range from 50 to 70 kcps (~ 0.6 × 10^5^ cps on this graph)

Figure 7 shows the DTCF curves as a function of the observed count rates for the two photopeaks and the full spectrum. When considering all counts in the PW, at the count rate of 4 × 10^4^, the DTCF values for the PW_113_ and PW_208_ were approximately 0.72 and 0.52 times lower, respectively, than those determined from the entire spectrum for the same count rate in the PW. However, when only primary photons are considered, the DTCF for the PW_113_ and PW_208_ were 3.6 and 2.6 times higher than those for the full spectrum.

In our imaging studies of patients being treated with 7.4 GBq of ^177^Lu and scans performed within 4 h after injection, typical count rates in the entire spectrum were at the level of 50–70 kcps, in the 208-keV photopeak window were equal to about 6–8 kcps, and the count rates for the primary photons were almost always below 2 kcps. (Since in these acquisitions, only 208-keV photopeak was used, similar information for 113 keV is not available). We had a very few patients where these rates were higher (up to a factor of two) and in these cases applying DT corrections would be important. Assuming these count rates, the DTCF when using only primary photons should be below 5%, while if the DT correction is based on the full spectrum, the value of DTCF would be three to four times lower.

## Discussion

### Energy spectra at different count rates

At high count rates, the spectra collected by the camera (see Fig. 3) show a wide range of effects, caused by different physical phenomena, which contribute to the observed DT. The reduction of intensity of both photopeaks (i.e., reduction of counts in *PW*_113_ and *PW*_208_) as the count rates increase can be explained by two effects: (1) count losses due to the finite processing time of the camera electronics and (2) the pileup effects where two or more photons which reach the detector within a very short time interval are counted as a single photon with energy equal to the sum of the two photons’ energies. Although the count loses due to electronics are rather energy-independent, the pileup effect causes the intensity to decrease in low-energy part of the spectrum and increase in the high-energy part (Fig. 3).

These effects are clearly seen in Fig. 3. The intensity of the photopeaks relative to the background is the highest in the spectra corresponding to the lowest count rates. In the scatter windows, however, it is at the highest count rates that the relative intensity of counts is the highest. Due to their high intensity, photopeak windows lose relatively more counts than they gain. The effect is opposite in the other (background) regions, and this is why scatter windows show increased intensities at the high count rates. Detector 2 which was positioned closer to the source (with less attenuating material) experienced higher count rates shows much stronger pileup effect than detector 1. The pileup effects have also been observed in ^131^I study by Guy et al. [13], similar to those that we are detecting in ^177^Lu spectrum.

Comparing the spectra recorded by the two detectors, it can be seen that the intensity of counts in the 113-keV photopeak window is approximately 1.3 times higher in the spectrum recorded by detector 2 than that recorded by detector 1, while the same ratio for the 208-keV peak is only 1.1. This effect can be explained by the higher attenuation of photons recorded by detector 1 due to additional 10 cm of water between the source and the detector (the effect is stronger for 113 keV than for 208 keV). Similarly, the intensity of characteristic Pb X-rays (peak at approximately 70 keV) from the collimator with respect to the characteristic X-rays of ^177^Lu (peak at approximately 50 keV) is higher for detector 1 due to more attenuation of the 50-keV photons relative to those at 70 keV. As the energy of photons increases, the linear attenuation of water decreases thus the intensity of the higher photopeak (208 keV) is less affected.

### Deadtime values

The behavior of the curves representing observed vs. true count rates corresponding to both detectors presented in Figs. 4, 5, and 6 is similar but not exactly the same. Due to different attenuation and scatter conditions for the two detectors, the count rates measured by detector 1 and also DT were much lower than those for detector 2. Therefore, in the subsequent discussion only detector 2 will be considered.

The USW of the 113-keV photopeak as well as both LSW and USW of the 208-keV photopeak show negative values of DT for detector 2. The interpretation of this effect is that, although in principle, the camera DT should remove counts from the spectrum, the pileup effect counteracts loses related to limited electronics processing time and adds more photons to these scatter energy windows. Since the intensity of the spectrum at low energies is high (due to large contribution from scattered photons), the probability of two such low-energy photons simultaneously arriving at the detector and being detected as a single high-energy photon is also high. The role of the DT correction is to re-create the correct spectrum shape by removing pileup and compensating for the count loses due to limited processing time of electronics.

When TEW scatter correction is performed for both detectors (after correcting each energy window for its own DT), both detectors behave similarly (Fig. 5d and Fig. 6d). This shows that the effects of scatter and attenuation have been accurately accounted for.

The differences in DT values determined using counts in the photopeak windows and in the entire spectrum, although high, are not surprising. Silosky et al. [12] analyzing ^99m^Tc spectra also found a factor of 20 difference between the DT determined based on the entire spectrum and on the photopeak only. Guy et al. [13] in their study of ^131^I found discrepancy amounting to 20%. The main causes for these differences are the pileup effect and the scatter. While the DT in the entire spectrum corresponds to the count losses mostly due to the camera electronics, the PWs are additionally affected by the pileup effects. Due to energy summing, some photopeak photons are detected in a high-energy part of the spectrum (outside the photopeak window) which effectively decreases counts in PW and results in its higher DT.

After scatter correction, the DT effect is higher for the lower energy photopeak, PW113 (Fig. 7d), most likely due to the fact that high intensity of the low-energy photons in the spectrum provides more opportunity for them to be not processed by the electronics and to pileup as compared to the 208 keV peak.

Previous studies [18] suggest that better quantification of ^177^Lu activity is achieved when data from the PW_208_ is used for image reconstruction. The fact that we have observed less deadtime for the 208 keV photopeak provides additional support that it should be used for quantification of ^177^Lu*.*

Considering differences in DT determined using the full spectrum and the PW counts only, a method allowing user to determine the DT for the PW if the DT for the entire spectrum is known has been proposed [7, 21]. The method involves calculating the window fraction and relating it to DT estimated from the full spectrum *τ*_fs_.

Window fraction *w*_f_ is defined as a fraction of counts detected within the specified window, relative to the total number of counts recorded in the full energy spectrum. It has been proposed that the observed DT for the specified energy window *τ*_w_ should be related to the full spectrum deadtime by the following relation:

5 $$ {\tau}_w=\frac{\tau_{\mathrm{fs}}}{w_f^{\eta }} $$

where *η* is a positive constant. Cherry et al. [22] propose a value of *η* = 1, while Silosky et al. [12] report values of *η* = 1*.*4.

Window fractions for the different energy windows and for both detectors are presented in Table 2. These values were calculated using all the 25 time points and taking the average window fraction with its corresponding standard deviation. We then used Eq. 5 with the values of *η* = 1 and *η* = 1*.*4 and compared the resulting *τ*_w_ with those obtained in our experiments (Table 3). None of the DT values for the six energy windows was correctly predicted based on the measured full spectrum DT and the corresponding window fraction. Moreover, the model cannot predict negative values of DT so it does not take into account pileup effects. We therefore do not support the use of this method to estimate the DT for the PW if the DT for the entire spectrum is known.

**Table 2:** Window fractions (in percentage) for the two photopeaks and detectors

*W*_f_ [%]	Detector 1	Detector 2
LSW_113_ [%]	8.73 ± 0.10	8.06 ± 0.10
PW_113_ [%]	16.41 ± 0.18	19.24 ± 0.26
USW_113_ [%]	8.29 ± 0.22	8.13 ± 0.49
LSW_208_ [%]	8.43 ± 0.09	6.77 ± 0.41
PW_208_ [%]	12.18 ± 0.24	13.89 ± 0.15
USW_208_ [%]	0.69 ± 0.15	1.32 ± 0.51

**Table 3:** Values for *τ*_w_ obtained with Eq. 5 using values of *η* available in the literature. Window fractions obtained from detector 2 were used as it covered a larger range of count rates

	*η* = 1	*η* = 1.4	True *τ*_w_	*η* required to obtain true value of *τ*_w_
*τ*_*w*113_ [μs]	1.0	1.9	6.04 ± 0.23	2.1
*τ*_*w*208_ [μs]	1.4	3.0	4.79 ± 0.18	1.6

The last two columns show the value of *η* which would be required to correctly predict the DT value determined in our experiments

### Correcting patient studies for the DT effect

Based on the results of our experiments and the above discussion, it is recommended that the procedure to correct patient’s images for the deadtime should include the following steps:

A. Determination of camera DT

1. Perform phantom experiments to measure the count rate performance and the DTCF of the camera. Use the same collimator, photopeak window, and two scatter windows as will be used for patient acquisition. Set additional windows to cover the remaining parts of the spectrum. The data acquired in these windows (summed) will provide the full spectrum count rates. The scatter conditions of the phantom used in these experiments should as much as possible be representative to the patient population.
2. Determine the observed and the true (using extrapolation method) count rates for each energy window (PW, LSW, and USW) independently.
3. Use these true and observed count rates for each energy window and the TEW method to calculate the true and observed primary photon count rate.
4. Determine *τ* for the primary photons.
5. Calculate the camera DTCF to be applied for primary photons. Graph the DTCF versus observed full spectrum count rates (more detail is given below when we mention the approximations of the method).

B. Performing DT corrections for patient studies

1. Apply the same energy windows settings as were used in the phantom experiments described above. For each projection of the patient tomographic scan, determine the observed count rates for the full spectrum window. Calculate the mean observed full spectrum count rate by averaging these overall projections.
2. Using the observed average full spectrum count rate, read the DTCF corresponding to primary photons only from the graph prepared in section A point 5.
3. Apply the DTCF to the quantitatively reconstructed image.

In this study, we created a DTCF plot for a Siemens SymbiaT camera using a MELP collimator (Fig. 7). It can be used to correct for deadtime losses in patient study if the same energy windows as used in our study are employed (see Table 1).

The proposed method represents a relatively simple protocol to be applied for radiotherapy patients’ scans where the deadtime has been observed (or suspected). The technique is general and can be applied to any camera. However, DT values must be determined experimentally for any other camera, collimator, and energy window settings. Nevertheless, our experience (collaboration with Universite Laval and work on several Symbia cameras in Vancouver) shows that the values reported in this paper for Siemens SymbiaT can be used with other cameras of the same type.

However, please note that the proposed method is based on the following the approximations: It is assumed that the scatter conditions of the phantom represent those that will be present in the patient study.

1. It is assumed that the DT losses are the same in each projection; therefore, the DTCF can be applied to the reconstructed image.
2. Since count losses due to DT depend on the scatter characteristics of a particular patient (related to his/her body size), it is reasonable to expect that the DTCF should be analyzed as a function of observed count rates in the full spectrum as these will be the least affected by the size of the patient. This approach is being used in our method as described above.

In our opinion, the issue of DT correction requires further investigation using large number of dataset from patients with different body shapes and sizes to fully understand the interdependence between primary photons count losses and patient’s body shape, scatter, and primary photon count rates.

In is important to realize that even when high activities are administered in therapy procedures, the DT count losses are typically observed only in the first scan, done shortly after the injection. Based on our experience with therapy patients, the DT correction is typically less or much less than 10%. Since dosimetry calculations are based on the time-integrated activity, the change in total organ/tumor dose due to the DT correction is expected to be relatively small.

We estimated this effect considering a patient study where three scans were performed at 3, 24, and 72 h. In the unlikely case when the DT correction increased the organ activity measured at the first time point by 10%, the change in the cumulative activity value would depend on the method used to integrate the area under the time activity curve (TAC). Assuming analytical fit to the data points, such increase of activity at the first time point would create a “steeper” shape of the TAC, which will result in 5% lower total dose to the organ. If the curve integration was performed using a “trapezoid” approach for the first three time points and exponential tail, the total dose would increase by 0.6%. Therefore, in our opinion, the correction for DT in most cases is not significant and the approximations used here are appropriate. The effects associated with curve fitting play much more important role in this case.

## Conclusions

Deadtime measurements for the Siemens SymbiaT camera have been performed, and the resulting data were fitted to the paralyzable model. The deadtime values (*τ*) were determined for each detector using counts in the entire spectrum and in the two photopeak windows set around the 113- and 208-keV gamma emissions of ^177^Lu*.* In order to match the scatter correction used in quantitative imaging studies, counts corresponding to both photopeak windows were corrected for scatter using TEW scatter correction method (after each photopeak and scatter window has been individually corrected for its own DT value).

The paralyzable model proved to be appropriate for the range of studied count rates. The DT values and the corresponding DTCF determined using primary photons only were substantially higher than those obtained from the analysis of the entire spectrum. Moreover, the DT values determined in this study did not agree with those predicted by the window fraction models found in the literature. This was most likely because these models do not consider primary photons but use all photopeak counts (i.e., do not perform the subtraction of scatter presented here).

The results of this study suggest that deadtime corrections should be performed based on the estimates of DT losses of the primary photons only, using scatter-corrected photopeak window and not by using the deadtime determined from the full spectrum. Additionally, since the deadtime values are lower for the 208-keV photopeak, it is recommended that ^177^Lu quantification should be based on acquisition of the 208-keV photons. Finally, a general procedure that can be followed to correct patient images for deadtime is presented.

## Abbreviations

DEW: Dual energy window
DT: Deadtime
DTCFs: Deadtime correction factors
FOV: Field of view
LEHR: Low energy high resolution
LSW: Lower scatter window
ME: Medium energy
NETs: Neuroendocrine tumors
PP: Primary photons
PRRT: Peptide receptor radionuclide therapies
PW: Photopeak windows
TEW: Triple energy window
USW: Upper scatter window

## References

[1] Kwekkeboom DJ, de Herder WW, van Eijck CHJ (2010). Peptide receptor radionuclide therapy in patients with gastroenteropancreatic neuroendocrine tumors. Semin Nucl Med.

[2] Kam BLR, Teunissen JJM, Krenning EP, et al. Lutetium-labelled peptides for therapy of neuroendocrine tumours. Eur J Nucl Med Mol Imaging. 2012;39:S103–S112. 10.1007/s00259-011-2039-y PMC3304065 22388631

[3] Kashyap R, Hofman MS, Michael M (2015). Favourable outcomes of 177 Lu-octreotate peptide receptor chemoradionuclide therapy in patients with FDG-avid neuroendocrine tumours. Eur J Nucl Med.

[4] Beauregard J-M, Hofman MS, Kong G, Hicks RJ (2012). The tumour sink effect on the biodistribution of 68Ga-DOTA-octreotate: implications for peptide receptor radionuclide therapy. Eur J Nucl Med Mol Imaging.

[5] Stabin M. The case for patient-specific dosimetry in radionuclide therapy. Cancer Biother Radiopharm. 2008;23(3). 10.1089/cbr.2007.0445 18593360

[6] Strigari L, Konijnenberg M, Chiesa C, et al. The evidence base for the use of internal dosimetry in the clinical practice of molecular radiotherapy. Eur J Nucl Med Mol Imaging. 2014:1976–88. 10.1007/s00259-014-2824-5 24915892

[7] Arnold JE, Johnston AS, Pinsky SM (1974). The influence of true counting rate and the photopeak fraction of detected events on Anger camera deadtime. J Nucl Med.

[8] Sorenson J (1975). Deadtime characteristics of Anger cameras. J Nucl Med.

[9] Chiesa C, Negri A, Albertini C (2009). A practical dead time correction method in planar activity quantification for dosimetry during radionuclide therapy. Q J Nucl Med Mol Imaging.

[10] Delpon G, Ferrer L, Lisbona A (2002). Correction of count losses due to deadtime on a DST-XLi (SmVi-GE) camera during dosimetric studies in patients injected with iodine-131. Phys Med Biol.

[11] Guirado D, Ramírez JC, De la Vega JM, Vilches M, Lallena AM (2012). Quality control for system count rate performance with scatter in gamma cameras. Phys Medica.

[12] Silosky M, Johnson V, Beasley C, Kappadath SC (2013). Characterization of the count rate performance of modern gamma cameras. Med Phys.

[13] Guy MJ, Flux GD, Flower MA, Ott RJ, Papavasileiou P, Chittenden SJ (2000). Practical scatter-independent gamma camera dead-time correction foriodine-131. 2000 IEEE Nucl Sci Symp Conf Rec (Cat No00CH37149).

[14] Beauregard J-M, Hofman MS, Pereira JM, Eu P, Hicks RJ (2011). Quantitative (177)Lu SPECT (QSPECT) imaging using a commercially available SPECT/CT system. Cancer Imaging.

[15] Celler A, Piwowarska-Bilska H, Shcherbinin S, Uribe C, Mikolajczak R, Birkenfeld B (2014). Evaluation of dead-time corrections for post-radionuclide-therapy (177)Lu quantitative imaging with low-energy high-resolution collimators. Nucl Med Commun.

[16] de Nijs R, Lagerburg V, Klausen TL, Holm S (2014). Improving quantitative dosimetry in 177Lu-DOTATATE SPECT by energy window-based scatter corrections. Nucl Med Commun.

[17] Sanders JC, Kuwert T, Hornegger J, Ritt P. Quantitative SPECT/CT imaging of 177Lu with in vivo validation in patients undergoing peptide receptor radionuclide therapy. Mol Imaging Biol. 2015;4:585–93. 10.1007/s11307-014-0806-4 25475521

[18] Ljungberg M, Celler A, Konijnenberg MW, Eckerman KF, Dewaraja YK, Sjogreen Gleisner K. MIRD pamphlet no. 26: joint EANM/MIRD guidelines for quantitative 177Lu SPECT applied for dosimetry of radiopharmaceutical therapy. J Nucl Med. 2015;9881(26) 10.2967/jnumed.115.159012 26471692

[19] Adams R, Hine GJ, Zimmerman CD (1978). Deadtime measurements in scintillation cameras under scatter conditions simulating quantitative nuclear cardiography. J Nucl Med.

[20] Uribe CF, Esquinas PL, Tanguay J (2017). Accuracy of 177Lu activity quantification in SPECT imaging: a phantom study. EJNMMI Phys.

[21] Zasadny KR, Koral KF, Swailem FM (2005). Dead time of an Anger camera in dual-energy-window-acquisition mode. Med Phys.

[22] Cherry SR, Sorenson J, Phelps ME, Methe BM (2004). Physics in nuclear medicine.

